# Postal survey methodology to assess patient satisfaction in a suburban emergency medical services system: an observational study

**DOI:** 10.1186/1471-227X-7-5

**Published:** 2007-06-15

**Authors:** Aaron W Bernard, Christopher J Lindsell, Daniel A Handel, Lindsey Collett, Paul Gallo, Kevin D Kaiser, Donald Locasto

**Affiliations:** 1 Department of Emergency Medicine, University of Cincinnati College of Medicine, Cincinnati, Ohio, USA; 2 Center for Policy and Research in Emergency Medicine, Oregon Health and Sciences University, Portland, Oregon, USA; 3 City of Reading Fire Department, Reading, Ohio, USA

## Abstract

**Background:**

Patient satisfaction is of growing importance to providers of emergency medical services (EMS). Prior reports of patient satisfaction have frequently used resource-intensive telephone follow-up to assess satisfaction. We determine the feasibility of using a single mailing, anonymous postal survey methodology for collecting patient satisfaction data from a suburban EMS system.

**Methods:**

Patients transported between January 2001 and December 2004 were mailed a brief satisfaction questionnaire. The questionnaire was printed on a pre-addressed, postage paid postcard and consisted of five questions that used a five-point Likert scale to assess satisfaction with EMS personnel and services provided. Three open-ended questions assessed concerns, the most important service provided, and methods for improving service. Survey response rate was the primary outcome of interest. The Chi-square test was used to compare rates between years.

**Results:**

The survey required about 6 man hours and cost about $70 per month. Overall response rate was 32.0% (857/2764; 95CI 30.3% – 33.9%). During the first year, response rate was 42.6% (95CI 38.5% – 46.8%), but was significantly lower in subsequent years (29.0% in year 2, 30.8% in year 3, and 27.6% in year 4, p < 0.05). There were 847/851 respondents (99.5%) who were satisfied or very satisfied with their EMS experience. Three patients felt the service was adequate and one was very unsatisfied. Open-ended questions suggested that interpersonal communications were the single most important contributor to patient satisfaction. Patients also reported that response times and technical aspects of care were important to them.

**Conclusion:**

Postal surveys for assessing patient satisfaction following EMS transport can achieve comparable response rates to similar surveys in other health care settings. Response rates did not decline after the second year of patient surveys, suggesting some stability after the initial year. Interpersonal communication was determined to be the single most important contributor to patient satisfaction.

## Background

Objective information to assure the quality of care delivered by EMS systems is in demand by governmental agencies, insurance companies, and customers [1]. Standard quality indicators such as response time and outcome data may not reflect everything that patients consider important. Patient satisfaction is a quality indicator that has the potential to provide valuable information about the care delivered by an EMS system. This indicator is considered an important marker of quality by paramedics [2].

Previous reporting of patient satisfaction has involved large urban EMS systems [3,4] and hospital-based systems [5]. While these reports highlight aspects of care with potential for improvement, the findings have limited generalizability to smaller systems within the United States. Further, these studies of patient satisfaction have generally used a telephone survey approach with high resource requirements [3]. To initiate patient satisfaction measures in smaller suburban and rural EMS systems, a methodology requiring less resources is necessary. A brief postal survey approach may satisfy this need. Research that evaluates the feasibility of this method is needed before any implementation can be recommended. To date, insufficient work has been completed on this topic.

Only one report was found that described the use of postal surveys for assessing patient satisfaction with EMS systems [4]. While that study showed a response rate of between 35% and 40% could be achieved using postal surveys, it was not conducted in the United States and, because of cultural differences, differences in prehospital care, and differences in the healthcare system [6], it is unknown if similar response rates will be achieved within a United States EMS system to appropriately assess patient satisfaction. We sought to evaluate the resources required to undertake, and the response rate to, a single-mailing anonymous postal survey of patient satisfaction in a small, suburban EMS system. In addition, we evaluate how the response rate varies over a four year period. We hypothesized that we would obtain a similar response rate to that observed in the one prior postal survey (35% – 40%), and that this response rate remains stable over time.

## Methods

### Design

This was an observational study of response to a postal survey of patient satisfaction that was instituted as part of a quality improvement program in a local EMS system in 2001. This study was approved by the University of Cincinnati Institutional Review Board.

### Setting

We evaluated the patient satisfaction survey distributed by Reading Fire and Rescue, Reading, Ohio. The city of Reading consists of 11,292 residents and approximately 1,200 EMS runs are carried out per year. The 2000 U.S. Census data indicates that the population is 93.7% white and 3.2% African American. The median per-capita income for the city is $23,527. The patient satisfaction survey was mailed to patients who were evaluated and transported between January 1, 2001 and December 31, 2004. Patients were not sent a survey if they were nursing home residents, were dead on arrival, had sustained cardiac arrest, had no mailing address or were known to be homeless. Patients with multiple runs during a single survey mailing period were sent only one satisfaction survey.

### EMS system

The EMS system in use at Reading Fire and Rescue is a paramedic first responder system, i.e. the first personnel on the scene are Emergency Medical Technicians – Paramedic (EMT-P). After the EMT-P crew assesses the patient, they determine whether transport to a hospital is required and, if so, by what level of care; patients can be transported by EMT-P, or by EMT-Basics, or a combination of these. Patient acuity is used to determine the combination of EMS personnel used for transport.

### Patient satisfaction survey

**Figure 1:**
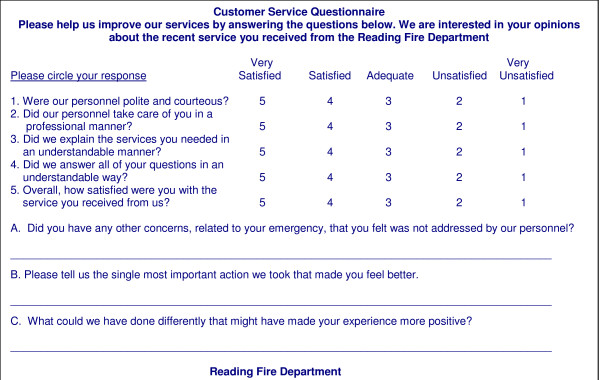
Patient satisfaction survey questionnaire mailed to eligible patients between January 2001 and December 2004.

The survey instrument used is shown in Figure 1. The survey was designed to be brief and to assess two primary domains of satisfaction: interaction and communication, in addition to overall satisfaction. The emphasis on interactions and communication was based on previous EMS-based research highlighting problems in this area [7,8]. Five quantitative questions were included that used a standard 5-point Likert scale, anchored by 'very satisfied' and 'very unsatisfied'. Two of the questions assessed personal interactions between EMS providers and patients, two assessed communication, and the fifth was a global satisfaction measure. (Figure 1). In addition, three qualitative questions were included to provide patients an opportunity to express concerns about care, suggestions for improvement, and to identify the most important factor affecting how the patient felt. Open ended questions also allow assessment of domains incompletely captured by structured questions, and can result in higher reports of elements of care that are dissatisfiers [9].

The patient satisfaction survey methodology was designed to be simple to implement on a routine basis, and to require minimal resources to conduct. The survey questions were printed on the back of a postage-paid, pre-addressed postcard, and the postcard was mailed to potential responders. An anonymous methodology was selected; this maximizes the likelihood of patients reporting dissatisfaction or problems [10], and can improve response rates [11]. The benefits of increased reporting of problems that can be addressed was considered to outweigh the benefits of being able to assess response bias.

Surveys were printed at a local shop in batches of 1000 as needed. The fire department lieutenant was responsible for getting the printing done. The lieutenant was also responsible for labeling and mailing of the surveys. This was done once every month. All patients or, for patients aged less than 18 years, their guardians, served by the EMS system during the previous month were identified from an electronic database that is used to capture run information. Names and addresses were printed on labels and mailed using the United States Postal Service. Neither the time from the run to the mailing, nor from the mailing to response was assessed. Completed surveys that were mailed back to the fire department were collated, interpreted, and reported by the lieutenant as needed for the purposes of the quality improvement program.

### Data management and analysis

Returned satisfaction surveys were provided to the investigators identified only by year of service. A summary description of patients served by the EMS system was also provided to contextualize results. Survey data were entered into Microsoft Excel (Microsoft Corporation, Redmond, WA) for subsequent analysis using SPSS v 13.0 (SPSS Inc., Chicago, IL). Open ended questions were categorized independently by two physicians familiar with the EMS system. The qualitative question relating to concerns was categorized as no answer, no concerns, and concerns noted. The question relating to the most important action to improvement how the patient felt was coded as no answer, interpersonal communication, response time, technical care, or some other action. The question pertaining to what could have been done differently was coded as no answer, nothing, or an identifiable change could be made. Cohen's kappa was used to assess the agreement in coding between the two physicians. In the case of disagreement, the most conservative (i.e. negative) response was allocated.

Response rates were computed as simple proportions and the 95% confidence intervals (95CI) of the proportions were computed using the score method. Response rates were compared between years using the Chi-square test. Secondary analysis was conducted to estimate the cost of conducting this patient satisfaction survey. The fire department lieutenant was interviewed to determine man hours spent on the quality improvement program. He was questioned as to hours he spent per month printing, labeling and mailing the survey. He was also questioned as to the hours he spent collating, interpreting, and reporting the results of returned surveys. The physical cost of each survey was determined by a combination of postal rates during the time of the study and printing costs.

## Results

### Main results

**Figure 2:**
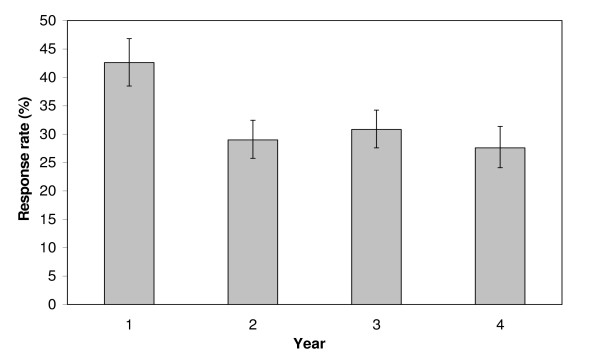
Response rates for each of the four years of the patient satisfaction survey; 95% confidence intervals of the response rates are shown.

The characteristics of patients served by the EMS system are given in Table 1, stratified by year. Of the 4,806 runs conducted during the four year period, 2,674 met criteria for mailing a patient satisfaction survey (Table 2). Of the 2,674 surveys mailed during the four year period, 857 were returned; the overall response rate was 32.0% (95CI 30.3% – 33.9%). The response rate during the first year was higher than in all other years (p < 0.002), but did not differ between years 2, 3 and 4 (p > 0.191) (Figure 2)

**Table 1:** Descriptive characteristics of patients served by the EMS system for each year of the study.

	**Year 1**	**Year 2**	**Year 3**	**Year 4**
**Gender**				
Male	55%	56%	56%	55%
Female	45%	44%	44%	45%
**Age**				
Mean	50	48	50	49
Range	0–103	0–93	0–98	0–95

**Response Time **(Minutes)	3:27	3:30	3:52	4:05

**Reason for Consultation**				
Injury	20%	19%	20%	19%
General Illness	15%	16%	15%	17%
Musculoskeletal	11%	10%	11%	12%
Cardiovascular	11%	10%	11%	10%
Respiratory	10%	9%	10%	8%
Psychiatric	7%	10%	6%	7%
Neurovascular	6%	5%	7%	5%
No cause for concern	5%	5%	5%	7%
Digestive	5%	6%	5%	4%
Metabolic	3%	2%	3%	2%
Other	1%	1%	2%	2%
Allergic Reaction	1%	1%	1%	1%
Genitourinary	1%	1%	1%	1%
OB/GYN	1%	1%	1%	2%
Arrest	1%	1%	1%	1%
Poisoning	1%	1%	1%	<1%
Environmental	1%	1%	<1%	<1%
Cancer	<1%	1%	<1%	1%
Hematologic	<1%	<1%	0%	0%
Infectious Disease	0%	<1%	<1%	0%

**Table 2:** The number of runs, number of patients excluded, and number of surveys mailed. Percentages are in brackets and referenced to number of runs, with the exception of surveys returned which are referenced to surveys mailed.

	**Number of runs**	**Patients not transported**	**Nursing home patients**	**Cardiac arrest/Dead on arrival**	**Repeat customer/No mailing address**	**Surveys mailed**	**Surveys returned**
**Year 1**	1,159	299(26)	58(5)	32(3)	209(18)	561(48)	239(43)
**Year 2**	1,196	318(27)	48(4)	31(3)	64(5)	735(61)	213(29)
**Year 3**	1,218	269(22)	48(4)	26(2)	106(9)	769(63)	237(31)
**Year 4**	1,233	272(22)	25(2)	31(3)	296(24)	609(49)	168(28)

**Total**	4,806	1,158(24)	179(4)	120(2)	675(14)	2,674(56)	857(32)

### Secondary results

To assess resource requirements, we estimated the cost and time resources hours necessary to routinely implement the survey and collect survey returns. Surveys were printed in batches of 1000, at a cost of $240.00 per batch or $0.24 per postcard. When combined with an outgoing and return postage rate of $0.37 per postcard, the total cost per survey mailed was $0.98. About 60–80 surveys were mailed each month, with the lieutenant reporting 2 hours to print and label these postcards. About 20 to 30 postcards were returned each month, with the lieutenant reporting about 4 hours spent collating, interpreting, and reporting. Thus, the overall requirements on a monthly basis was about $70 and 6 hours.

For comparison to other systems, and to assess the face validity of the survey instrument, we also analyzed the patient satisfaction data. Table 3 shows the proportions of patients who were satisfied or very satisfied with the care they received. Throughout the four year period, 847/851 respondents (99.5%) were satisfied or very satisfied on the overall satisfaction measure (question 5). Three patients felt the service was adequate and one was very unsatisfied. Agreement between the two physicians coding the open ended questions was high (Kappa > 0.964). The results are shown in Table 4. Three percent of respondents reported that concerns they had were not addressed by our personnel. Interpersonal communication was the single most important action that made patients feel better, followed by response time and technical actions. Six percent of patients reported that something could have been done differently to make the experience more positive.

**Table 3:** Proportion and 95% confidence interval of the proportion of respondents indicating they were satisfied or very satisfied with assessed service components. The number responding to each question is shown.

	**Year 1**		**Year 2**		**Year 3**		**Year 4**	
Question 1 (n = 853)	100.0	(98.0 – 100.0)	100.0	(97.8 – 100.0)	100.0	(98.0 – 100.0)	99.4	(96.2 – 100.0)
Question 2 (n = 852)	100.0	(98.0 – 100.0)	99.5	(97.0 – 100.0)	99.1	(96.6 – 99.9)	99.4	(96.2 – 100.0)
Question 3 (n = 842)	98.7	(96.0 – 99.7)	98.1	(94.8 – 99.4)	99.1	(96.6 – 99.8)	98.2	(94.4 – 99.5)
Question 4 (n = 838)	99.1	(96.6 – 99.9)	99.5	(96.9 – 100.0)	99.6	(97.2 – 100.0)	98.8	(95.2 – 99.8)
Question 5 (n = 848)	99.6	(97.3 – 100.0)	100.0	(97.8 – 100.0)	99.1	(96.6 – 99.9)	99.4	(96.2 – 100.0)

**Table 4:** Responses to the open ended questions.

	**Response category**	**Proportion**	**95% confidence interval**
**QuestionA**: Did you have any other concerns, related to your emergency, that you felt was not addressed by our personnel?	No answer	33.7	(30.6 – 37.0)
	No concerns	63.0	(59.6 – 66.2)
	Some concerns	3.3	(2.2 – 4.8)

**Question B**: Please tell us the single most important action we took that made you fell better.	No answer	22.8	(20.1 – 25.8)
	Interpersonal communication	42.3	(38.9 – 45.7)
	Response time	21.2	(18.5 – 24.1)
	Technical actions	10.4	(8.5 – 12.7)
	Other actions	3.3	(2.2 – 4.8)

**Question C**: What could we have done differently that might have made your experience more positive?	No answer	41.0	(37.7 – 44.4)
	Nothing	52.8	(49.3 – 56.1)
	Something	6.2	(4.7 – 8.1)

## Discussion

This research demonstrates that a low-resource postal survey can achieve consistent response rates of about 30% for assessing patient satisfaction following EMS transport in a small suburban setting, at a cost of $70 and 6 hrs per month. Our early response rate of 43% is similar to comparable reports [4,12-15]. Postal satisfaction surveys mailed to hospitalized patients after discharge have been reported to have response rates from 41% to 58% [12,13]. A response rate of 42% was found in a postal patient satisfaction survey of outpatient practices [14]. Kuisma et al. demonstrated response rates between 36% and 40% to postal patient satisfaction surveys in a large urban EMS system in Finland [4]. The more consistent response rate of about 30% achieved beyond the first year of our study is higher than achieved for postal surveys following emergency department visits; the response rate to one postal satisfaction survey of emergency department patients was 19.7% [15].

There are two major advantages to postal surveys over telephone surveys for measuring patient satisfaction: a reduced resource burden [12,16] and anonymity for reporting sensitive issues; a bias towards more favorable opinions when using telephone methodology has been reported [13,17]. However, these advantages are balanced by the lower response rate frequently observed for postal surveys when compared to telephone surveys; observed response rate to a telephone-based EMS patient satisfaction survey was 49% [3].

Many techniques are available to maximize postal survey response rates, although some of these require increase resource allocation. Recent systematic reviews of postal surveys have characterized the odds of response that can be expected when individual techniques are used [16,18]. We employed two techniques that have been shown to increase response rate: a short questionnaire and a stamped return envelope [18]. Other techniques that can improve response rates we have not implemented include the use of follow up telephone calls (1.5 times increased odds of response), use of colored inks, contact prior to delivery, or repeat mailings (1.4 times increased odds of response), and personalized questions (1.2 times increased odds of response) [18].

The decision to utilize these additional techniques is a balance of response rate, response bias, accuracy of responses and resource allocation. The response rate achieved by our methodology appears to be comparable to similar patient satisfaction surveys and likely represents a baseline rate, but this could be increased using some of the above techniques. For example, a 1.4 increased odds of response would increase our response to about 40%, while a 1.54 increase in odds would result in a response rate of about 58%. The addition of colored inks is unlikely to contribute much to the overall resource burden, except in printing costs. Contact prior to delivery and follow up telephone calls would be more resource intensive; telephone satisfaction surveys are about 10%–20% more costly than postal satisfaction surveys, and postal surveys with telephone follow up are similar in cost to telephone surveys [11]. The use of personalized questions would not be possible under the current anonymous methodology.

To the best of our knowledge, our EMS satisfaction survey is the first to report changes in response rate over time. The response rate during the first year was significantly higher than in all other years. If generalizable to other settings, it is likely that response rates during an initial period of monitoring satisfaction may be an overestimate of the likely response rate achievable in a longer term quality program that incorporates patient satisfaction measures. The lack of change in response rates during the latter three years of our study suggests that some stability in response rates may have been achieved after the first year. Reasons for, and implications of, such a pattern of response to patient satisfaction surveys have yet to be explored and represent an area for future work.

In addition to demonstrating the response rates to a postal satisfaction survey, our data show a high degree of patient satisfaction with the care received in (Table 3). The degree of patient satisfaction is sufficiently high to warrant concerns about the usefulness of the questions asked. The quantitative questions were not based on a previously validated questionnaire but were based on previous research in the field. Research on complaints against EMS systems has identified rude and unprofessional conduct by EMS professionals as the most common reason for complaint [7,8]. We expect that these complaints would have been reported in response to questions 1 and 2. Further, we elected the postal methodology to maximize honesty in reporting dissatisfaction or problems; satisfaction tends to be reported as higher in telephone or face-to-face surveys than in anonymous postal surveys due to a tendency to provide socially acceptable responses. Two EMS systems that have reported patient satisfaction by means of telephone interview reported high satisfaction [3,5]. A postal satisfaction survey done in a large EMS system found high satisfaction as well [4]. These consistent findings of high satisfaction support our results.

In response to qualitative questions, only 3.3% of patients reported that they had concerns that were not addressed by the EMS personnel (Table 3). Furthermore, 94% of patients did not feel anything could have been done differently to make their experience more positive. One interesting finding was that patients often used the space provided for qualitative responses to express gratitude for service instead of answering the question. This was similarly observed by Persee et al. [3], and suggests it may be important to provide space for such unsolicited commentary in a satisfaction survey.

Patients reported that the single most important action which made them feel better was interpersonal communication. When this question was asked in a large urban EMS system, the answer received most frequently received was "quick response" [3]. This highlights the differences between EMS systems serving different populations, and the need for data from different settings. Interpersonal communication as an important part of patient satisfaction is highlighted in other research including reports of complaints made to EMS systems [4,5,7,8]. Not surprisingly, interpersonal communication is also reported to be an important part of patient satisfaction in the emergency department [19].

There were two domains of patient satisfaction not included in our survey but which were frequently included in qualitative responses: response time and technical ability. These were intentionally excluded. Patients perception of response time has been demonstrated to be inaccurate [20], and our quality program monitors response time objectively. Actual response times during the study period are listed in Table 1. There also exist quality indicators to monitor technical ability that is not best gauged using patient perspectives. For instance, outcome measurements such as prehospital cardiac arrest data using the Utstein style are used in our quality program [21]. While we objectively monitor quality of response time and technical ability, we have yet to consider how patient expectations within these domain impacts satisfaction.

Our secondary results are limited but their value to our quality improvement program is evident in the changes we have made to education of our EMS providers. For example, prior to reviewing these quality data, interpersonal communication was not emphasized in continued education yet this was found to be the most frequent factor impacting patient's satisfaction. Thus, we now assign a portion of our continued education time to this topic. Through a variety of didactic lectures and simulation sessions, we are attempting to refine skills in this area to meet the needs of our patients. While it is recognized only limited information can be provided in response to brief questions such as those used in our survey, the information can be used to identify factors impacting patient satisfaction, and thus guide the development of education that focuses on those domains important to patients. The overall high satisfaction achieved is taken with the limitations of the study, including the potential for responder bias, but still provides some reassurance of the quality of our daily work.

### Limitations

While we have shown that implementing an anonymous, postal satisfaction survey is feasible for a small suburban EMS system, and that satisfaction with this system is high, several limitations are evident that must be considered when interpreting results. The primary limitation is a low response rate, despite its similarity to satisfaction surveys, and the unknown response bias. To the best of our knowledge, no study has addressed the characteristics of non-responders to EMS patient satisfaction surveys. However, there is evidence to suggestion that in the United States, non-responders in patient satisfaction surveys tend to be minorities and have lower socioeconomic status than responders [22]; it is possible that the views of these vulnerable population are not captured in our data. In addition, there is evidence that non-responders show a systematic trend towards lower satisfaction than responders [23]. This would suggest that the high satisfaction reported in this study is likely an overestimate of true satisfaction

The ability of our study to demonstrate stability of response rate over time is limited as well. Our study is longer than prior reports and did find stability in response rate in the latter years. Further research is needed to see if this trend is reproducible and continues over longer periods of time.

Interpretation of secondary results is also limited by the survey methodology. Anonymous reporting prevents consideration of the type of run or type of responder as a modifier of satisfaction. Previous studies suggest that certain types of calls are associated with less patient satisfaction [4]. Our survey also did not assess patient satisfaction for non-transported patients, the decision to transport or not may also impact satisfaction; one analysis of non-transported patients suggest that they are more satisfied with their care than transported patients [4].

Finally, our assessment of feasibility is based on response rates and crude estimates of cost. The determination of whether or not a satisfaction survey is feasible requires a balance between achieving an acceptable response rate with acceptable expenditure. In our case, we achieved response rates similar to those of other satisfaction surveys, with a 'minimal' expenditure, and thus for our system we concluded the satisfaction was feasible.

### Recommendations

This study is, to our knowledge, the first to report on the feasibility and response rates than might be expected in anonymous, postal patient satisfaction surveys for small, suburban EMS systems. Our findings are likely to be generalizable to similar systems, and replication of our methods might allow others to obtain satisfaction data and, more importantly, identify dissatisfiers that should be addressed within their system.

This study raises several important questions that must be answered in future research. First, the questions incorporated in the survey were not developed in a systematic manner but were designed to obtain information on specific factors known to affect satisfaction with EMS services. Development of a validated, EMS-specific patient satisfaction survey tool that captures all of the domains impacting patient satisfaction would greatly enhance the ability of EMS systems to measure and improve satisfaction.

Secondly, research to minimize response bias in satisfaction surveys of EMS services should be attempted. For example, further research is needed to determine if low-resource methods, such as the use of colored inks, can improve response. We would currently recommend that response rates similar to telephone surveys, about 50%, should be the goal of future work with postal surveys. In addition to research on methods for improving response rate, a greater understanding of the bias involved in non-response is needed. For example, we demonstrated that response rate might decrease after the first year of measuring satisfaction. The reasons for this might uncover new biases not previously considered.

Future research should also examine variation in satisfaction with run type, with type of responder, and with the decision to transport or not transport. These questions will require non-anonymous methods and represent an area for future study.

Once appropriate methods are in place to measure and monitor satisfaction, and factors impacting satisfaction with EMS services are well elucidated, it will be possible to implement interventions aimed at improving satisfaction. We have already begun thus by incorporating training on interaction and communication within our system. The systematic study of such interventions could provide evidence for maximizing satisfaction with care.

## Conclusion

Anonymous, single-mailing, postal surveys can achieve response rates of about 30% for assessing patient satisfaction following EMS transport with a cost of about $70 and 6 hrs a month. Response rates may be artificially high during initiation of the satisfaction assessment process, but they appear to remain stable during the following time period. Interpersonal communication is an important contributor to patient satisfaction, and should be considered when training EMS personnel to improve patient satisfaction.

## Competing interests

The author(s) declare that they have no competing interests.

## Authors' contributions

AWB conceived and designed the study. LC and PG collected the data. DAH and AWB interpreted qualitative responses. CJL conducted data analysis. AWB, DAH, CJL, LC, KDK, and DL contributed to the interpretation of the analysis. AWB drafted the manuscript and all authors contributed significantly to its content and provided critical review. AWB takes responsibility for the work as a whole. All authors read and approved the final manuscript.

## Pre-publication history

The pre-publication history for this paper can be accessed here:


